# Hypertension approaches and recent SGLT2i studies

**DOI:** 10.1111/1753-0407.70034

**Published:** 2024-11-25

**Authors:** Zachary Bloomgarden

Several recent studies remind us to more fully address hypertension, suggest potentially useful approaches, point out the particular benefit of sodium‐glucose cotransporter 2 inhibitors (SGLT2i), and indicate other effects of these agents.

In a randomized controlled trial (RCT) of 12 821 hypertensive persons with type 2 diabetes (T2D) having cardiovascular disease (CVD), or chronic kidney disease (CKD), or with 2 or more CVD risk factors, assigned to systolic blood pressure (SBP) goals of 120 vs 140, SBP at 1 year was 121.6 vs 133.2 mmHg, respectively, with the composite endpoint of stroke, myocardial infarction, heart failure or CV death occurring 21% less frequently in those assigned to the lower SBP target. Stroke and new onset albuminuria were reduced 21% and 13%, respectively, and similar benefit was seen regardless of age, sex, prior CVD or CKD, HbA1c, or duration of diabetes or of hypertension.^1^

In a meta‐analysis of seven hypertension (HBP) trials lasting 3.1–4.7 years, comparing SBP goals of <130 versus ≥130 mmHg among 72 138 participants with a mean age of 62–68, achieveing an SBP of <130 mm Hg was associated with a reduction in major CVD risk by 22%, mortality by 11%, stroke by 26%, coronary heart disease (CHD), including myocardial infarction, by 17%, heart failure (HF) by 31%, and CV mortality by 27%. In four trials, randomization to SBP <120 versus <140 reduced CVD 18%, mortality 15%, stroke 19%, CHD 13%, HF 24%, and CV mortality 28%. Rates of hypotension, syncope, injurious falls, electrolyte abnormalities, and acute kidney injury were higher with lower BP targets, but these events remained infrequent, with the number needed to harm 508 for hypotension and 3222 for electrolyte abnormality. Subgroup outcomes were reported inconsistently, so that differences in outcome could not be ascertained by diabetes, age, or CVD risk.^2^

Given these results, it is sobering to look at achieved levels of BP control. In the combined dataset from The National Health and Nutrition Examination Survey (NHANES) of 2017–2018 and 2019–2020, among 7328 persons age ≥18, 3129 had SBP ≥130 or diastolic BP (DBP) ≥80, of whom 58% were unaware of HBP. Only 825 had a history of HBP with SBP <130. Of those with known HBP, 18% had an SBP of 130–139 or DBP of 80–89 with low atherosclerotic cardiovascular disease (ASCVD) risk, meeting the criteria for lifestyle modification, but 82% met criteria for lifestyle plus additional medication, with 26% having a history of CVD, 21% having CVD, and 21% having diabetes. NHANES is designed to allow estimates of the US population, showing 120 million people in the United States with HBP, 35 million of whom meet the criteria for additional antihypertensive medication, which the authors suggest “may reflect the slow adoption of the updated guidelines.”^3^

Similarly, analysis of NHANES 2021‐2023 showed that 47.7% of adults had hypertension, of whom 51.2% were treated with antihypertensive medicine and only 20.7% had achieved BP < 130/80.^4^

An interesting study analyzed optimal patient‐positioning for BP measurement. The protocol was carefully defined as follows: “Participants walked for 2 minutes to replicate a typical clinical scenario before arriving at a BP measurement station. They then underwent a 5‐minute seated rest period with their back and feet supported…in a quiet and private space, and participants were asked not to talk to researchers or use their smartphones during BP measurements.” Three BP readings were taken in each of three positions, with the arm resting on a desk, with the arm held by the side, and with the arm resting on the lap. Measurements were spaced 30 s apart, using an upper‐arm cuff selected based on the participant's measured mid‐upper arm circumference. The authors found that SBP/DBP levels were significantly different: 126/74 mmHg for the desk position, 130/78 mmHg for the lap position, and 133/78 mmHg for the side position. The potential explanations include increase in distance when the arm is positioned below the heart leading to increase in brachial artery hydrostatic pressure, lower arm position decreasing venous return causing compensatory vasoconstriction with increase in vascular resistance, and muscle contraction by the unsupported arm causing transient increased BP.^5^ The implication is that, particularly with more aggressive HBP goals, proper BP measurement is crucial.

Given the increasing emphasis on greater intensive of HBP treatment, renal denervation seems to be an intriguing approach, with a meta‐analysis of 10 trials involving 2478 patients with HBP, using radiofrequency, ultrasound, or alcohol‐mediated catheter ablation versus sham controls, reporting a 24‐h and office SBP reduction of 4.4 and 6.6 mmHg, respectively, and a 24‐h and office DBP reduction of 2.6 mmHg,^6^ comparable to the 5.1‐mmHg SBP lowering of individual antihypertensive agents studied in the Blood Pressure Lowering Treatment Trialists' Collaboration.^7^

It is similarly instructive to assess the antihypertensive efficacy of SGLT2i. Among 12 960 patients at Kaiser Permanente Southern California with HBP who started SGLT2i, mean age 64.5 years, 42.1% female, 95.5% with T2D, and 16.9% with HF, mean SBP was reduced from 133.9 to 128.6 and DBP 70.8 to 68.3 mmHg, for 5.3 and 2.5 mmHg reductions, respectively. Among 2913 patients with proteinuria, SBP decreased from 140.0 to 133.9, and among 3774 patients with treatment‐resistant hypertension SBP decreased from 136.8 to 130.6 mmHg,^8^ suggesting that SGLT2i should be considered as effective antihypertensive agents to add to our armamentarium. These results are similar to those of an earlier metanalysis of the ambulatory BP‐lowering effect of SGLT2i, in which 3.6 and 1.7 mmHg reductions in SBP and DBP respectively were seen, with reductions comparable to those with low‐dose hydrochlorothiazide.^9^ SGLT2i should therefore be considered antihypertensive agents in addition their glycemic and cardio‐ and nephroprotective effects. The BP‐lowering effect of glucagon‐like peptide‐1 receptor agonists (GLP‐1RA) appeared more modest in a recent meta‐analysis, showing SBP decreases of 3.4 mmHg with semaglutide, 2.6 with liraglutide, 1.5 with dulaglutide, and 3.4 with exenatide, particularly in studies with longer treatment duration, correlating with the degree of reduction in HbA1c and in body weight.^10^

Several recent reports add further to our understanding of effects of SGLT2i. In a population‐based study of people starting treatment with these agents, among 20 146 people with T2D and pre‐existing nephrolithiasis, 14 400 initiated an SGLT‐2 inhibitor and 5746 a GLP‐1RA. In total, 1924 of the former and 853 of the latter had a recurrence of nephrolithiasis, with an adjusted rate ratio of 0.67 and number needed to treat (NNT) of 20. In a similar analysis comparing SGLT2i with DPP4i initiators, the nephrolithiasis rate ratio associated with SGLT‐2i initiation was 0.73. Among those not having a history of nephrolithiasis, 1072 new nephrolithiasis events occurred among 112 728 weighted initiators of an SGLT‐2i (7.4 per 1000 person years) and 499 new events among 41 623 weighted initiators of a GLP‐1RA (12.8 per 1000 person years), with an adjusted rate ratio of 0.58. A mechanism of nephrolithiasis benefit might be increasing urinary volume and reducing the concentration of stone forming solutes, as well as increasing urinary pH enhancing uric acid solubility. In the same analysis, 4409 patients also had baseline history of gout, of whom 3159 initiated an SGLT‐2i and 1250 a GLP‐1RA, with recurrent gout events in 161 and 71, respectively, corresponding to a rate ratio of 0.72. SGLT2i lower uric acid, with a potential mechanism being the competition of glucose with urate for transporter mediated reabsorption in the proximal tubule, enhancing uric acid excretion.^11^

SGLT2i may also play a role in treatment of the syndrome of inappropriate antidiuresis (SIAD). A RCT of 88 hospitalized persons with SIAD and serum sodium (Na) <130 mEq/L who were given 25 mg of empagliflozin daily for 4 days, with fluid restriction of <1000 mL/day, led to a Na increase of 10 versus 7 mEq/L, with increased urine osmolality, albeit with two cases (4.6%) of overly rapid hyponatremia correction and four cases (9.3%) of transient decrease in renal function. A crossover RCT of 14 outpatients with chronic SIAD and median Na 131 mEq/L showed a median Na increase of 4.1 mEq/L compared to placebo, also with increase in urine osmolality.^12^

One caveat: A recent meta‐analysis of 51 RCTs with data from 97 589 patients addressed the risk of amputation associated with SGLT2i. Although occurring in <2% of treated patients, SGLT2i were associated with a 47% increase in overall risk, and a 68% increased risk in studies with treatment duration >100 weeks, albeit with moderate heterogeneity.^13^

## DISCLOSURE

The author has nothing to report.

## References

[1] Bi Y , Li M , Liu Y , et al. Intensive Blood‐pressure control in patients with type 2 diabetes. New Engl J Med. 2024. doi:10.1056/NEJMoa2412006 39555827

[2] Whelton PK , O'Connell S , Mills KT , He J . Optimal antihypertensive systolic blood pressure: a systematic review and meta‐analysis. Hypertension. 2024;81(11):2329‐2339. 39263736 10.1161/HYPERTENSIONAHA.124.23597 PMC11483200

[3] Richardson LC , Vaughan AS , Wright JS , Coronado F . Examining the hypertension control Cascade in adults with uncontrolled hypertension in the US. JAMA Netw Open. 2024;7(9):e2431997. 39259543 10.1001/jamanetworkopen.2024.31997 PMC11391330

[4] Fryar CD , Kit B , Carroll MD , Afful J . Hypertension prevalence, awareness, treatment, and control among adults age 18 and older: United States, August 2021–August 2023. NCHS Data Brief 2024;511. 10.15620/cdc/164016 PMC13655856 40085792

[5] Liu H , Zhao D , Sabit A , et al. Arm position and blood pressure readings: the ARMS crossover randomized clinical trial. JAMA Intern Med. 2024;e245213. doi:10.1001/jamainternmed.2024.5213 39373998 PMC11459360

[6] Vukadinović D , Lauder L , Kandzari DE , et al. Effects of catheter‐based renal denervation in hypertension: a systematic review and meta‐analysis. Circulation. 2024;150:1599‐1611. doi:10.1161/CIRCULATIONAHA.124.069709 PMC11560572 39355923

[7] Nazarzadeh M , Canoy D , Bidel Z , et al. The blood pressure lowering treatment Trialists' collaboration: methodological clarifications of recent reports. J Hypertens. 2022;40(5):847‐852. 35221323 10.1097/HJH.0000000000003107

[8] An J , Sim JJ , Zhou MM , et al. Blood pressure reduction and changes in antihypertensive medication use among patients with hypertension who initiated sodium‐glucose Cotransporter‐2 inhibitors. J Clin Hypertens (Greenwich). 2024;26:1318‐1321. doi:10.1111/jch.14915 PMC11555531 39373635

[9] Georgianos PI , Agarwal R . Ambulatory. Blood pressure reduction with SGLT‐2 inhibitors: dose‐response meta‐analysis and comparative evaluation with low‐dose hydrochlorothiazide. Diabetes Care. 2019;42(4):693–700. 30894383 10.2337/dc18-2207 PMC6429633

[10] Rivera FB , Lumbang GNO , Gaid DRM , et al. Glucagon‐like peptide‐1 receptor agonists modestly reduced blood pressure among patients with and without diabetes mellitus: a meta‐analysis and meta‐regression. Diabetes Obes Metab. 2024;26(6):2209‐2228. 38505997 10.1111/dom.15529

[11] McCormick N , Yokose C , Lu N , et al. Comparative effectiveness of sodium‐glucose cotransporter‐2 inhibitors for recurrent nephrolithiasis among patients with pre‐existing nephrolithiasis or gout: target trial emulation studies. BMJ. 2024;30(387):e080035. 10.1136/bmj-2024-080035 PMC11524131 39477370

[12] Tzoulis P . Empagliflozin: a wonder drug for the treatment of SIAD? Front Endocrinol (Lausanne). 2024;15:1453159. 39435353 10.3389/fendo.2024.1453159 PMC11491318

[13] Geng L , Sun B , Chen Y . A meta‐analysis of randomized controlled studies examining the effects of sodium‐glucose co‐transporter‐2 inhibitors on peripheral artery disease and risk of amputations. Diabetes Obes Metab. 2024;26(11):5376‐5389. 39267269 10.1111/dom.15901

